# Treatment of Diphtheria

**Published:** 1893-12-30

**Authors:** 


### LONDON HOSPITAL.

### Treatment of Diphtheria.

Since all patients suffering from this disease are, whenever practicable, sent to one of the Metropolitan Asylums Board hospitals, the treatment of the disease at the London Hospital is limited to the not inconsiderable number of cases that arrive at the hospital in such a condition that it is not considered advisable to subject them to the additional exertion and delay entailed by removal to a fever hospital.

Almost without exception the patients are young children, and in the greater number the disease has already involved the larynx, so as to cause dangerous obstruction to breathing.

Owing to the serious nature of the cases admitted, the first question that arises, and one that often has to be settled within a very short period from admission is that of tracheotomy, and in coming to a decision on this important and vital question the chief points considered are the following:—

*The Age.*—Very rarely is tracheotomy successful in saving life in infants under a year old, when the disease has already reached such a point that it is causing dangerous obstruction to respiration.

*Collapse.*—When a child is already in a state of collapse, livid, with feeble, irregular pulse, suffering from the effects of the diphtheritic poison on the organism, whether or not there be much tracheal obstruction, tracheotomy is not advisable.

*The State of the Lungs.*—When there is much broncho-pneumonia present, as evidenced by patches of crepitant räles here and there over the chest, with perhaps louder moist sounds of congestion in addition, tracheotomy, thought it may relieve the obstruction in the windpipe, cannot have any marked influence on the state of the lungs, and if the amount of efficient lung available for respiration is not enough for the oxygenation of the blood, tracheotomy is therefore useless. In determining the amount of lung block that would counter-indicate operation, the age is, of course, taken into consideration, an infant withstanding far less than a child of nine or ten; and in all cases where there is doubt as to whether or not the child could recover if the obstruction in the windpipe was relieved, tracheotomy is, if possible, performed.

The indications for operation are mainly the presence of considerable obstruction to breathing, as evidenced by lividity, quickened respiration, and sinking in of the suprasternal and supra-clavicular spaces, with marked retraction of the epigastrium and lower ribs, to an extent not accounted for by the amount of broncho-pneumonia, or other disease of the lung itself, allowance being made for the amount of congestion that will result from the continuance of tracheal obstruction for any length of time.

Turning now to the general hygiene of the patient. The child is isolated in a small special ward kept at a temperature of from 60 deg. to 70 deg., the bed covered in on three sides and over the top, for the double reason of warding off draughts and keeping the air in the immediate neighbourhood of the child moist, the latter being effected by a steam kettle, the long spout of which plays a jet of steam into the cot.

In a state of health a child almost invariably breathes through the nose, unless there is some obstruction in the nasal passages; and in the passage through the nostrils the air becomes almost saturated with moisture, but whenever any serious obstruction to breathing is present, it matters not in what portion of the respiratory tract, the mouth is used for the admission of air, which then reaches the lungs neither sufficiently heated nor containing its proper amount of moisture. The object then of the steam is to bring the inspired air as near as possible to the state in which it would reach the lungs under normal conditions, and this is more especially necessary when respiration is being carried on through a tracheotomy tube, straight from the outer air to the lungs.

The feeding is of great importance in such a debilitating disease as diphtheria, and consists chiefly of milk and eggs, in various preparations and combinations, meat essences and extracts, with usually some form of alcohol, either spirit or wine.

A very troublesome complication occasionally arises giving some difficulty in feeding; this is the return of fluids through the tracheotomy wound or tube. Its real cause is debated, but apparently has no relation to the common form of post-diphtheritic paralysis, occurring much earlier in the course of the disease, and usually passing off within a week. Solids and semi-solids can be swallowed without difficulty or return through the wound. The food while this condition persists has to consist entirely of solids and semi-solids. The milk is given thickened with cornflour, arrowroot, or gelatine. Meat essence as a jelly. Stimulants can be given in the same way by being added in measured quantities to jelly or gelatine when liquid, so that when set and cut up in a given number of equal parts, each piece will contain the necessary dose of alcohol. It is advisable to give food frequently, as the appetite is always impaired, and only a little may be taken at a time. Attention to this point is specially necessary at night. In a bad case some nourishment is given at not longer intervals than every four hours, even if the patient is asleep. The internal treatment most used consists of iron, usually the perchloride, in full doses, with quinine, and where there is evidence of broncho-pneumonia combined with some expectorant as squill, or some stimulant expectorants such as carbonate of ammonia, the aromatic spirit of ammonia, or ether, may be substituted for it.

Locally, the greatest reliance is placed on keeping the fauces clean, without trying to peel off or forcibly remove the membrane. This is accomplished by swabbing the fauces with a solution of boric acid, permanganate of potash, or weak solution of chlorinated soda. Often, in addition, the patches of membrane are painted with perchloride of iron and glycerine—one part of the liq. ferri perchlor. fort. to three parts of glycerine—or the parts are sprayed with quinine solution (three grains to the ounce) or perchloride of mercury 1 in 20,000. Many other local applications have been tried, such as papain, hydrochloric, and lactic acids, but without any marked success, perhaps owing somewhat to the fact that the cases are all from the first of great severity.

A very large proportion of the cases admitted require tracheotomy, an anæsthetic, chloroform being almost always used, the opening in the trachea being in the two and three rings below the cricoid, though in children the cricoid is also sometimes cut. A fair sized opening in the trachea is an advantage, rendering the insertion of the tracheotomy tube much easier, and at the time of operation the edges of the tracheal wound being held apart by the dilating forceps, a good view of the inside of the trachea is obtained. For this purpose light can be reflected into the wound with a mirror, a laryngoscope reflector, especially the form where the mirror is attached to a band round the forehead, being the most useful, as it can be tilted so as to throw the light in and yet leave the hands of the operator free. If there is much membrane spreading down the trachea the prognosis is proportionately bad. At the time of operation the membrane is removed through the wound whenever possible; often a strip or strips can be removed with dressing forceps or a feather; sometimes a clean rubber catheter attached to a syringe is passed through the wound into the trachea, and some membrane may be drawn into the eye by suction with the syringe. If the precaution is not taken of looking to see the condition of the trachea, the tracheotomy tube may be pushed into a collection of membrane, or may push a strip in front of it, blocking the end of the tube and preventing respiration through it, necessitating its removal and reinsertion.

The most useful forms of tube are the Durhams and Parkers, both of which are found to fit better than the ordinary tubes, the vertical portion of which is at right angles to the horizontal. The direction of the trachea is downwards and backwards, so a tube that forms a right angle cannot fit perfectly. Durham's tube fits well because the vertical portion is very short, and the length of the horizontal part can be varied by means of the shield, which is moveable. The Parker's tube is bent at an angle that fits the trachea better, and when made with a moveable shield, so that the length of the tube can be varied according to the depth of the wound, fits better than any other variety. The bivalve tube is never used: Morrant Baker's rubber tubes only occasionally.

The wound is dusted with iodoform, and kept as clean as possible, a piece of lint soaked in carbolised oil or covered with boracic ointment being kept over it, with an opening for the tracheotomy tube. The inner tube requires to be kept clear from mucous and membrane, for which purpose a feather dipped in limewater, or a solution of either bicarbonate of soda or potash is the most useful, and is frequently removed and cleaned with one of these solutions or hot salt and water.

A few layers of gauze, or a thin moist sponge placed over the tube, assists in keeping the air that is entering the lungs moist, a steam kettle being always kept going close to the patient, and the room being maintained at a uniform temperature between 65 deg. to 70 deg. The outer tube is changed on the second or third day for cleaning, and permanently removed from the fourth to the fifth day onwards, according to the persistence of the membrane and the amount of block in the air passages above the tube; it is removed gradually, that is kept out for an increasing time every succeeding day. The wound closes rapidly by granulation. Sometimes a child will not breathe when the tube is removed; this may be due to nervousness, especially when the tube has been in use a longer time than usual, and the habit of breathing through it well established. Cautious daily removal of the tube nearly always overcomes this difficulty, or a Gresswell's tube is substituted for the one previously in use. This tube is in shape like the ordinary pattern, but at the part where the tube is angled, and therefore when in place inside the trachea there is a hole through both inner and outer tubes, so that respiration can still take place freely through the larynx. The outer end of the tube projects more than usual, and is perforated by several lateral holes; a cap screws over this end, and can be gradually screwed down so as to block the lateral holes as breathing is carried on more and more through the larynx, till when the cap is screwed down tight no air at all enters through the tube, which can then be permanently removed. Very rarely a cicatricial band in the trachea requires division before the tube can be left off.

The wound itself sometimes takes on a sloughy and inflamed condition. The best application under these circumstances is found to be the lotio plumbi, a piece of lint constantly wet with the lotion being kept over the wound and adjacent skin. In cases where there is threatening cardiac failure, as shown by increasing irregularity of pulse, hypodermic injections of strychnine and digitaline are used, and children take these drugs well and react quickly to them, or some ammonia and ether with tincture of digitalis or strophanthus is given by mouth, with an increased amount of alcohol. Needless to add, a constant watch is kept on the patient, so that the tube shall not become blocked or covered over. During convalescence watch is kept for the onset of post-diphtheritic paralysis, of which the earliest signs are the loss of the patella reflex and return of fluids through the nostrils on swallowing, showing paralysis of the soft palate. For this condition the drug found most efficacious is strychnine, either as the liquor strychniæ or as the extract or tincture of nux vomica, combined with iron and other tonics for the co-existing anæmia; a liberal dietary in addition being all-important.

